# Smoking, smoking cessation and otorhinolaryngologists in the state of Sao Paulo, Brazil

**DOI:** 10.1016/S1808-8694(15)30040-9

**Published:** 2015-10-19

**Authors:** Aracy Pereira Silveira Balbani, Jair Cortez Montovani, Lidia Raquel de Carvalho

**Affiliations:** a Voluntary Professor, PhD from the Department of Otolaryngology-Head and Neck Surgery - Medical School of Botucatu, Paulista State University (UNESP); b Associate Professor of Otolaryngology/Head and Neck Surgery – Medical School of Botucatu – Paulista State University (UNESP); c Assistant Professor – Department of Biostatistics – Biosciences Institute – Paulista State University (UNESP) Department of Otolaryngology-Head and Neck and Neck Surgery – Medical School of Botucatu – Paulista State University (UNESP)

**Keywords:** smoking, nicotine, tobacco use disorder, tobacco use cessation, bupropion, medical education

## Abstract

Otorhinolaryngologists are directly involved in the diagnosis and management of smoking related diseases, including upper airway malignancy. It is important that the specialists have skills to treat smoking and nicotine dependence. It is also known that there are smokers amongst doctors.

**Aim:**

To assess the opinions and practices of the otorhinolaryngologists of the state of Sao Paulo, Brazil, concerning smoking and nicotine dependence, and evaluation of smoking habits of the specialists.

**Study Design:**

Crosssectional.

**Materials and Methods:**

We randomly selected 600 otorhinolaryngologists of Sao Paulo State, Brazil. A survey was mailed to the specialists in March 2005. We gathered data received from March to May 2005.

**Results:**

There were 209 respondents. Forty-seven specialists (46.4%) rated themselves as moderately familiar with the methods for treatment of nicotine dependence, and 60 (28.7%) as unsatisfactorily familiar. One hundred and forty-four respondents (68.9%) have never smoked, 50 (23.9%) were former-smokers, nine (4.3%) were occasional smokers and six (2.9%) were regular smokers.

**Conclusion:**

The prevalence of smoking in the sample of 209 otorhinolaryngologists of Sao Paulo State, Brazil, was 7.1%.

## INTRODUCTION

Smoking is considered one of the most severe pandemics that has ever plagued human kind. In this context, otolaryngologists are directly involved in the diagnosis and treatment of diseases caused by smoking, including upper air way cancer.

It is important that the physician be properly prepared along medical school and medical residency to approach and treat smoking and nicotine chemical addiction. Notwithstanding, a study carried out in medical schools of 159 countries showed that only 11% of those had disciplines that specifically deals with smoking; 64%, on nicotine chemical addiction and 30%, on smoke quitting techniques^2^.

On the other hand, we know that there is a reasonable number of smokers among physicians themselves. In Brazil, sampling investigations during scientific meetings from 1970 to 1991 showed that the ratio of smoking doctors varies between 20 and 50%^3^.

Mirra, Rosemberg (1997)^3^ questioned 23% of Brazilian physicians and observed about 6.4% of smoking doctors, with a prevalence in the age range of 35 to 69 years, without significant difference between genders. The largest amount of smokers was seen in medical specialties in which there is not much direct contact with patients: clinical genetics, hospital management and forensic medicine. Among 320 otolaryngologists included in the study, 4.3% smoked.

Considering how important it is to treat smoking and nicotine chemical addiction, this paper aims at: a) investigate opinions and approaches of São Paulo State otolaryngologists about this topic; and b) assess smoking habits among the specialists.

## MATERIALS E METHODS

600 São Paulo State Otolaryngologists were randomly selected from the Brazilian Association of Otolaryngology- Head and Neck Surgery register. On March, 2005, These physicians received, by mail, a standard questionnaire (attached) with questions about:

a) Professional profile (gender, age, race, year of graduation, working area – General ENT or subspecialty);
b) Opinion about the teaching of nicotine dependence during otolaryngology residency;
c) “Usually physicians advise their patients to quit smoking. Do you believe that if the patient knew that the physician him/herself smoked could impact the treatment?”;
d) Approach to help nicotine addicted patients to quit smoking (advice, use of bupropion, nicotine chewing gum, nicotine patches, acupuncture, referral to a clinician, other);
e) Familiarity with the means to treat nicotine addiction;
f) Smoking habit. Classification criteria of the physicians’ smoking habit were: a) never smoked; b) former smoker (has quit smoking for at least 6 months now); c) occasional smoker (misses a day of smoking for a least 6 months) and d) smoker (smokes daily for at least 6 months)^4^.

To former smokers we asked how they managed to quit smoking and if seeing patients with head and neck cancer influenced that decision.

To smokers and occasional smokers we asked: tobacco use profile (age at which they started smoking, type of tobacco they use, daily consumption, etc.), positive and negative aspects of smoking in their lives, attempts made towards quitting, their habit of performing exams for lung and larynx cancer, and if they suffer any sort of prejudice because they smoke.

Their identification in answering the questionnaire was optional.

The answers received from March to May, 2005 underwent statistical analysis by the chi-squared test through the SAS 6.12 version computer software, using 5% as significant level (p<0.05).

## RESULTS

Of the 600 questionnaires sent, 209 (34.8%) were properly filled out and sent back. 76 participants (36.4%) chose to identify themselves and 133 (63.6%) chose to remain anonymous. There was no relationship between answer anonymity and the smoking habit (p=0.192).

a) Participants’ profile

Among the 209 participant, 147 were male (70.3%) and 62 were female (29.7%); 198 were white (94.7%) and 11 were Asian (5.3%), with ages varying between 25 and 77 years (average of 44.4 years). Six physicians did not inform their age.

129 Otolaryngologists (62%) had been graduated for over 15 years (p=0.001); 38 (18.1%) between 11-15 years; 28 (13.3%) between 6-10 years and 13 (6.1%) less than 5 years. One physician did not inform his graduation year (0.5%).

9 different working areas for these professionals were mentioned, the most frequent were: General Otolaryngology (157 professionals, 75.1%), Otology (14 professionals, 6.7%), Pediatric Otolaryngology (nine professionals 4.3%), Head and Neck Surgery and Rhinology (seven professionals in each subspecialty – 3.3%).

b) Opinion on the teaching of nicotine dependence during otolaryngology residency.

**Chart 1:**
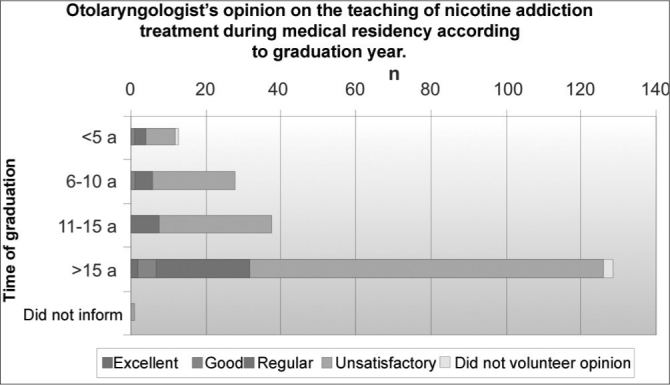
Opinion of otolaryngologists (n=209) on the teaching of nicotine addiction treatment during otolaryngology residency.

This teaching was deemed poor by 155 specialists (74.2%; p=0.001), regular by 41 (19.6%), good by seven (3.3%) and excellent by two (1%). Four specialists (1.9%) did not give their opinion. The opinion distribution versus years after graduation is depicted on Chart 1.

Attachment **Standard questionnaire about smoking sent to the otolaryngologists.**

1. Gender [ ] female [ ] male

2. Age: years

3. Race [ ] white [ ] black [ ] yellow

4. Year of graduation:

5. Main area of work

[ ] General otolaryngology [ ] Laryngology and voice [ ] Rhinology [ ] Otology

[ ] Head and Neck surgery [ ] Pediatric otolaryngology [ ] Other:

6. What is your opinion on the quality of teaching about the treatment of nicotine addiction during otolaryngology medical residency?

[ ] excellent [ ] good [ ] regular [ ] not satisfactory

7. Physicians usually advise patients to quit smoking. Do you believe that if the patient knew that the physician him/herself smoked would impact the treatment?

[ ] no

[ ] yes, negative impact (the physician loses authority/credibility)

[ ] yes, positive impact (the patient believe the smoker physician understands better the difficulty of smoke quitting)

8. What is your approach for the addictive patient to quit smoking? (More than one answer is possible)

[ ] counseling [ ] prescribes bupropion [ ] refers to acupuncture

[ ] prescribes nicotine chewing gum [ ] prescribes nicotine skin patches

[ ] refers the patient to the clinician [ ] other:

9. What is your opinion on the efficacy of the following in nicotine addiction treatment?

**Table:** 

	very efficient	little efficient	inefficient
smoker patient education	[ ]	[ ]	[ ]
bupropion use	[ ]	[ ]	[ ]
nicotine chewing gum use	[ ]	[ ]	[ ]
nicotine skin patches use	[ ]	[ ]	[ ]
acupuncture	[ ]	[ ]	[ ]
psychotherapy	[ ]	[ ]	[ ]

10. What is your level of familiarity with nicotine addiction treatment methods?

[ ] excellent [ ] good [ ] regular [ ] unsatisfactory

11. What is your smoking habit?

[ ] never smoked

[ ] former smoker (has quit smoking for at least 6 months)

[ ] occasional smoker (can refrain from smoking for one day for at least 6 months)

[ ] smoker (smokes daily for at least 6 months)

IF THE PHYSICIAN IS A FORMER SMOKER, PLEASE ANSWER

12. How did you manage to stop smoking? (More than one answer is possible)

[ ] by his/her own means [ ] by order of a medical colleague

[ ] for health reasons [ ] used bupropion [ ] underwent acupuncture

[ ] used nicotine chewing gum [ ] used nicotine skin patch [ ] other:

13. Seeing head and neck cancer patients has influenced your decision?

[ ] yes [ ] no

IF THE PHYSICIAN IS AN OCCASIONAL SMOKER OR A SMOKER, PLEASE ANSWER:

14. Age in which you started smoking:

15. Type of tobacco you use

[ ] “common” cigarette [ ] low tar varieties [ ] stogie [ ] cigar [ ] pipe

16. Consumption: cigarettes/day

17. Inhale the smoke? [ ] yes [ ] no

18. When do you smoke the most?

[ ] during the week [ ] on the weekends [ ] it doesn’t matter

[ ] when alone [ ] when with company [ ] it doesn’t matter

[ ] at home [ ] at places of leisure [ ] it doesn’t matter

19. The physician smokes in the office, clinic or hospitals?

[ ] no

[ ] yes, Where? [ ] only at designated facilities [ ] even out of designated facilities

20. Mention smoking aspects that you consider positive in your life

21. Mention smoking aspects that you consider negative in your life

22. Have you ever tried to stop smoking?

[ ] no

[ ] yes. Number of attempts: Maximum withdrawal time:

Most unpleasant withdrawal signs:

23. Do you consider yourself a nicotine addicted? [ ] no [ ] yes

24. Do you undergo larynx cancer prevention exams?

[ ] no

[ ] yes, annually [ ] yes, every six months

[ ] yes, but only when some symptom arises. Which?

25. Do you undergo lung cancer prevention exams?

[ ] no

[ ] yes, annually [ ] yes, every six months

[ ] yes, but only when some symptom arises. Which?

26. Do you feel prejudiced for being a smoker? (more than one answer possible) [ ] no

[ ] yes, by patients [ ] yes, by family members

[ ] yes, by non-smoker medical colleagues [ ] yes in meeting other people

27. Open space for comments about smoking.

c) “Usually physicians advise patients to quit smoking. Do you believe that if the patient knew the physician him/herself smoked it could impact the treatment?”

Of those who participated in the study, 193 (92.3%) said such fact would have a negative impact (“the physician loses credibility/authority”); four (2%) said it would have a positive impact (“the patient believes the smoking physician understands better the difficulty in quitting smoking”) and 11 (5.2%) said it would not impact the treatment. One physician (0.5%) did not give his opinion.

d) Approach for the nicotine addicted patient to stop smoking.

32 different approaches to manage nicotine addiction were reported. Forty-six otolaryngologists (22%) advise their patients and refer them to clinicians, usually the pneumologist; 44 (21.1%) advise and prescribe bupropion; 40 (19.1%) only advise their patients; nine (4.3%) advise their patients and refer the patient to psychotherapy or smoke quitting support groups; seven (3.3%) advise and refer the patient to acupuncture; three (1.4%) advise their patients and refer them to the psychiatrist.

Three specialists (1.4%) mentioned that they educate their patients in regards of sports practice and physical activities, and one refers the smoker to “Relaxing Techniques such as Yoga”. One physician prescribes phytotherapy ( raw garlic during meals, oatmeal tea, calendula flower gargling, chew clove when craving, inhaling black pepper to reduce withdrawal symptoms).

Thirteen otolaryngologists (6.2%) did not mention counseling as an approach to nicotine addiction.

Analyzing smoke quitting pharmacotherapy, 72 professionals (34.4%) prescribe bupropion and 28 (13.4%) indicate nicotine replacement therapy (NRT) – nicotine chewing gum or skin patches – and 15 (7.1%) prescribe bupropion and NRT concurrently. One otolaryngologist prescribes nortriptyline.

The Otolaryngologists’ opinion on the efficacy of counseling, bupropion, nicotine chewing gums/skin patches, acupuncture and psychotherapy in nicotine addiction management are seen on Table 1. Nine physicians (4.3%) stated that the efficacy in the nicotine addiction treatment is directly related to the patients motivation to stop smoking.

**Chart 2:**
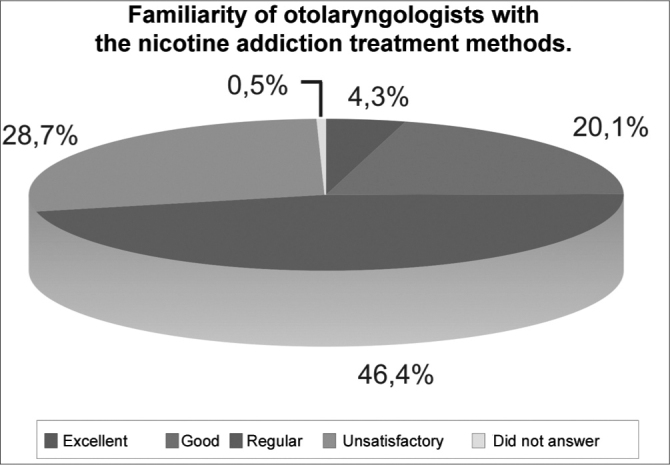
Familiarity of otolaryngologists with the nicotine addiction treatment methods (n=209).

e) As far as familiarity with the means used to manage nicotine addiction is concerned, seen on Chart 2, 97 otolaryngologists (46.4%) consider it regular (p=0.001) and 60 (28.7%), poor. One specialist did not answer.

There was not relation between the specialist's familiarity with the means of treatment and the time of graduation (p=0.281).

f) Smoking habit of the participants.

144 otolaryngologists (68.9%) never smoked (p=0.001); 50 (23.9%) were former smokers; nine (4.3%) were occasional smokers and six (2.9%) were regular smokers.

**Chart 3:**
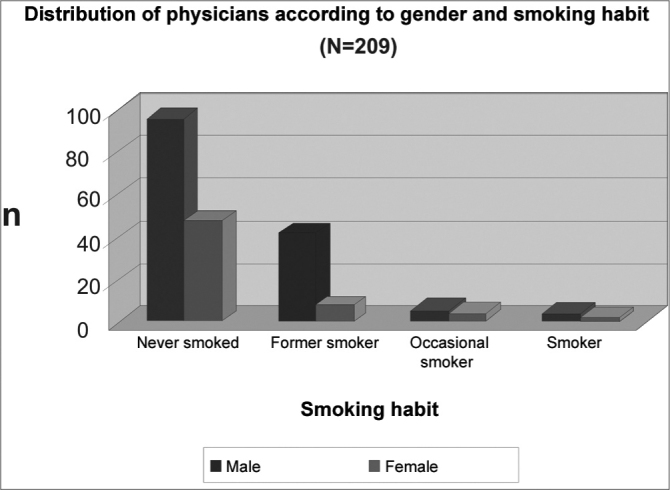
Distribution of participant frequence according to smoking habit and gender. X2=6,92, p=0,14

The distribution of the smoking habit frequence according to the participant's gender is seen on Chart 3. Among the 50 former smokers, 42 were men (84%) and eight were women (16%).

The average age of the smokers/occasional smokers and former smokers were 43.3 years and 52.9 years, respectively. Two former smokers did not state their ages.

Among the 15 smokers and occasional smokers, the age when they started to smoke varied between 11 and 22 years, with an average of 15.2 years. Seven (46.7%) smoked “regular” cigarettes; 5 (33.3%), the “low tar” varieties; 2 (13.3%) smoked cigarettes and cigars. One occasional smoker did not inform the type of tobacco he smoked.

The daily consumption varied between one and 20 cigarettes, with an average of 14 cigarettes/day among the smokers, and 2 cigarettes/day among occasional smokers. One occasional smoker did not report on the amount of tobacco used. Fourteen occasional smokers and regular smokers (93.3%) inhale, and one occasional smoker did not answer.

Nine smokers or occasional smokers (60%) do not smoke in their offices, clinics or hospitals. Five (33.3%) smoke in medical care facilities, however only in the allowed areas. One occasional smoker did not answer this question.

Of the 15 physicians who use tobacco, eight (53.3%) do not undergo tests for larynx cancer early detection and six (40%) do it annually. Nine (60%) do not undergo lung cancer prevention tests, four do it annually and one does it when coughing. On occasional smoker did not say if the undergoes respiratory tract cancer prevention exams.

Nine professionals feel prejudiced – by family, nonsmoking medical colleagues and other people, because they smoke, while five do not suffer any prejudice and one did not give his opinion. One otolaryngologist said that being a smoker is like being a “leper”.

Among smokers and occasional smokers, eight (53.3%) do not consider themselves nicotine addicted, six (40%) believe they are addicts and one did not volunteer his opinion.

Questioned about the positive aspects of smoking in their lives, the specialists said: anxiety soothing, the cigarette flavor and the pleasurable feeling. One otolaryngologist described the act of smoking as a “delicious vice”. Three smoking physicians stated that there are no positive aspects related to smoking.

As negative smoking-related aspects: foul odor brought about by the cigarette, halitosis, stomach discomfort, headache, sleep disorder and the possibility of chemical addiction. Three specialists stated that smoking sets a “bad example” or has “negative influence over the family”.

Eleven of the smokers or occasional smokers (73.3%) have already tried to stop smoking. The number of attempts to stop smoking varied from one to five, and one specialist stated that he is “constantly trying to quit smoking”. Three specialists have already tried to stop smoking and one did not answer this question.

The maximum time spent without smoking varied from one month to three years. The most unpleasant withdrawal symptoms were: anxiety (four cases – 36.4%), irritability (two cases – 18.2%), weight gain and smoking craving. One physician, who does not consider himself nicotine addicted – reported he did not show any withdrawal symptoms.

Of the 50 former smokers, 37 (74%) quit smoking on their own; five (10%) for health reasons and one (2%) for both reasons. Other reasons mentioned for quit smoking were: family pressure and past history of cigarette-related cancer in the otolaryngologist's family. For 27 former smokers (54%), seeing patients with head and neck cancer did not influence them on their quitting decision, while 20 (40%) felt influenced, and three (6%) did not answer. Three used bupropion and one used acupuncture in order to stop smoking.

ne otolaryngologist who smoked 2-3 packs/day and was able to quit smoking said he uses his own life experience as argument when he is counseling his smoking patients.

g) Opinion on smoking

We stress some comments from the participant in the study: “How to bring awareness to the asthmatic and smoking physician?” “A good doctor-patient relationship is the key for success” in order to quit smoking. “Treating smoking requires a multidisciplinary team”. “It is worthless to tell a smoker that smoking is bad; he already knows it. We have to convince him that quitting brings advantages and is good, in other words, don’t fight smoking, promote healthy habits.” “The quality of teaching about nicotine addiction should improve during otolaryngology residency”. Cigarettes are a “socially accepted drug that involves large profits to the tobacco industry and the government”. “The tobacco advertisement is very influential”.

Two otolaryngologists highlighted the tobacco alcohol consumption relationship with head and neck cancer, stressing that alcohol dependence also requires the help of a health professional. One specialist remembered that physicians should interfere equally about passive smoking.

## DISCUSSION

In our settings, otolaryngology residency programs highlight more the surgical treatment of tobacco-related neoplasms than nicotine addiction and smoke quitting. Thus, it is frequent that the resident follows patients treated by extensive surgeries (total laryngectomy with bilateral cervical lymph node resection, for instance), but he/she does not learn how to approach the issue of smoking quitting with their patients.

As a matter of fact, most specialists who participated in this study assessed in a negative way the teaching of nicotine addiction treatment during otolaryngology residency. For 74.2% of the interviewed ENTs, the teaching of this topic was not satisfactory.

This deficiency in medical teaching was reflected in the self-assessment of the professionals’ knowledge: 46.4% consider regular their familiarity with the management of nicotine dependence and 28.7%, consider it not satisfactory.

When facing a case of nicotine addiction, 22% of the otolaryngologists advise the patient to stop smoking and refer them to a clinician, while 21.1% advise the patient and prescribe bupropion, and 19.1% only advise the patients.

According to the Consensus from the National Cancer Institute (INCA) to approach and treat the smoker, drug therapy is indicated for the nicotine addicted patients^5^. Medical advice alone may not have the desired effect in these cases. On the other hand, 6.2% of the interviewed ENTs did not mention advising as the fundamental step for the smoker or nicotine addict to become aware and be motivated to stop smoking^5^.

We noticed that bupropion and nicotine replacement therapy (NRT), first line approaches used to treat nicotine dependence, are prescribed, respectively, by 34.4% and 13.4% of the specialists. Although literature data point towards a combined use of bupropion and NRT to doubled the smoke quitting success rates^6^, only 7.1% of the study participants used this practice. Nortriptyline, prescribed by one of the ENTs interviewed, is considered second line medication to treat nicotine addiction, because of its antidepressant and ansiolytic effect^6^.

As to the otolaryngologists’ opinion about the efficacy of nicotine addiction treatments, there was one relevant finding: for most specialists, counseling, drug therapy, acupuncture and psychotherapy are not very efficient. This opinion comes to reinforce the observations of 4.3% of the physicians about the patients’ motivation as a the decisive factor to quit smoking.

We observed a 7.1% prevalence of smokers in our sample of 209 otolaryngologists in the State of São Paulo. This rate is above the one found by Mirra; Rosemberg (1997)^3^, notwithstanding, it is much below what has been seen in some developed countries. According to the literature, 44% of Greek physicians are smokers, as well as 30% of Dutch, 33% of Danish^7^ and 34% of French^8^.

Many people ask why physicians smoke. What is expected from health care professionals is that they should not smoke, specially because thy have solid scientific knowledge about the maladies caused by smoking^9^.

Notwithstanding, Rosemberg (1988)^10^ reminds us that people usually start smoking as teenagers. As we could see in this paper, the average age in which the ENTs started smoking was 15.2 years, in other words, most already smoked before entering medical school. This finding is similar to that from Campos (1992)^11^, Campos (1993)^12^ and Mirra; Rosemberg (1997)^3^.

According to Pereira (1999)^9^, “being aware of scientific knowledge is one thing; changing one's habit based on this knowledge is something else”. Thus, even during or after the medical course, many professionals continue to smoke. There are many reasons for this: 1) smoking is seen as a normal social action^9^, 2) there may be nicotine addiction^9^ – manifested through a pleasurable feeling when smoking and unpleasant sensations in smoking withdrawal, 3) many medical school professors also smoke^2^ and 4) many smokers use cigarette smoking to alleviate anxiety in moments of stress^13^ – of which there are quite many in a physicians daily routine.

Following the trend of reduction in smoking habit in the general young population, we found among the 44 ENTs with less than 35 years of age who participated in the study, 42 individuals who had never smoked. This data constitutes a very positive horizon as to the health of the new generation of specialists.

It is interesting to notice that 92.3% of the ENTs who took part in this study stated that, once the patient knows that the physician himself is a smoker, this fact will have a negative impact on the smoke quitting treatment (“the physician loses authority/credibility”). One female otolaryngologist, former smoker, stated that she used to hide even from her family in order to smoke and used to tell her patients she did not smoke. This behavior of attempting to hide the physician's “bad example” or “incoherence” is similar to those found by Campos (1993)^12^ in a study with physicians from the Federal District. However, they differ from British medical schools students, of which 71% believed that smoking is a physician's free and personal choice, without any relation to his/her professional performance^2^.

The daily average consumption was of 14 cigarettes/day among smoker ENTs and 2 cigarettes/day among occasional smokers, data that coincide with those found by Campos (1992)^11^ and Campos (1993)^12^.

Just as in the work by Campos; Barra Sobrinho (1991)^14^, we noticed that most of the smoking physicians or occasional smokers (73.3%) have already tried to stop smoking.

We stress that the average ages of the total number of participants in the study and the former smokers were, respectively, 44.4 years and 52.9 years. Besides, 84% of former smokers were men. Therefore, there was a trend in that there is a greater ratio of former smokers among the elderly male physicians, as it has been already shown in other studies^8^, ^13^.

Most of the ENTs who stopped smoking (74%) did it on their own. Contrary to what was supposed, although seeing head and neck cancer patients is common in the ENT's daily practice, this fact did not influence the decision to stop smoking for 54% of former smokers.

Of the 15 smoker otolaryngologists who took part in our study, 53.3% do not undergo larynx cancer prevention exams and 60% do not undergo lung cancer prevention exams. We point out that some of the most important papers about the impact of smoking in mortality rate and cancer – published by Doll; Hill in 1954^15^, and by Doll et al. in 2005^16^ – were carried out exactly among British physicians. In other words, it is important to educate the smoker physician – without discriminating them – that he/she should also prevent smoking-caused diseases, and specially, quit smoking.

In conclusion, we deem necessary to enhance the teaching of nicotine addiction treatment in residency programs for otolaryngologists. In parallel to this, it is necessary to hold broad campaigns, aimed at the specialists, to educate and provide scientific information about smoking. The support from the specialty associations, medical schools and class entities is of paramount importance in achieving this goal.

## CONCLUSIONS

The study with 209 São Paulo State Otolaryngologists regarding smoking and nicotine addiction treatment revealed that:

a) the teaching of such topic in otolaryngology residence programs was not considered satisfactory by 74.2% of the participants;
b) 97 professionals (46.4%) assessed their familiarity with the treatment means as regular, 60 (28.7%) as not satisfactory and 47 (20.1%) as good;
c) facing nicotine addiction cases, the management used by 22% of the otolaryngologists is to advise the patients to quit smoking and refer them to clinical care, while 21.1% advise and prescribe bupropion, and 19.1% only advise;
d) bupropion and the nicotine replacement therapy, first line approaches to treat nicotine addiction, are prescribed by 34.4% and 13.4% of specialists, respectively;
e) counseling, bupropion, nicotine chewing gum, nicotine patches, acupuncture and psychotherapy were considered of little efficacy in treating nicotine addiction by: 54.5%, 46.4%, 45.5%, 51.2%, 45.9% and 45% of the specialists, respectively;
f) 144 otolaryngologists (68.9%) never smoked (p=0.001); 50 (23.9%) are former smokers; nine (4.3%) are occasional smokers and six (2.9%) are regular smokers;
g) the average age they started smoking was of 15.2 years; average daily consumption was of 14 cigarettes/day among smokers and 2 cigarettes/day among occasional smokers;
h) of the 15 smoker ENTs, 53.3% do not undergo any exam for larynx cancer prevention and 60% do not undergo exams to prevent lung cancer.

## THANKS

The authors thank the physicians who very kindly accepted to participate in the study, the Brazilian Association of Otolaryngology and Cervico-Facial Surgery for their support. We also thank Mrs. Vânia Rosa Moraes, Ms. Cinthia Scolastico Cecílio and Luciana Borragine de Oliveira, and employees from the Tatuí (SP) mail office for their practical help during our investigation.
